# Occurrence, Prevalence, and Distribution of Haemoparasites of Poultry in Sub-Saharan Africa: A Scoping Review

**DOI:** 10.3390/pathogens12070945

**Published:** 2023-07-17

**Authors:** Danisile Tembe, Mokgadi P. Malatji, Samson Mukaratirwa

**Affiliations:** 1 School of Life Sciences, College of Agriculture, Engineering and Science, University of KwaZulu-Natal, Durban 4001, South Africa; malatjim@ukzn.ac.za (M.P.M.);; 2 One Health Center for Zoonoses and Tropical Veterinary Medicine, Ross University School of Veterinary Medicine, Basseterre P.O. Box 344, Saint Kitts and Nevis

**Keywords:** haemoparasites, poultry, distribution, infection, co-infection, prevalence, sub-Saharan Africa

## Abstract

This review collated existing data on the occurrence, distribution, and prevalence of haemoparasites of poultry in sub-Saharan Africa. A literature search was conducted on three electronic search databases using search terms and Boolean operators (AND, OR). The results recorded 16 haemoparasites, viz., *Leucocytozoon* spp., *L. marchouxi, L. neavei, L. sabrazesi, L. schoutedeni, Haemoproteus columbae, H. pratasi, Haemoproteus* spp., *Plasmodium* spp., *P. gallinaceum, P. circumflexum, P. juxtanucleare, Trypanosoma avium, T. gallinarum, T. numidae,* and *Hepatozoon* spp. from a wide range of poultry species distributed across Nigeria, Kenya, South Africa, Tanzania, Uganda, Botswana, Zimbabwe, Ghana, Cameroon, and Zambia. Infections due to *Haemoproteus* and *Leucocytozoon* species were the most common and documented in eight of the ten reviewed countries. The presence of mixed infections was observed in quails, pigeons, chickens, ducks, turkeys, and guineafowls, but predominantly in chickens. Co-infections by *Plasmodium* spp. and *Haemoproteus* spp. were the most common, which may be attributed to the distribution of these species, coupled with the availability of vectors they are associated with in areas from which they were documented. The information generated in this review is essential for improving existing preventive and control measures of these parasites in sub-Saharan Africa.

## 1. Introduction

The poultry production industry holds an important position in the provision of animal-based protein to many communities worldwide through the provision of meat and eggs [1]. According to the Food and Agricultural Organization (FAO) of the United Nations [2], poultry farming provides a significant contribution to food, nutrition, and financial security throughout the world. In Africa, poultry meat represents approximately 25% of all meat types, and in certain parts of Africa it represents about 100% of the available sources of animal protein [3]. Apart from the nutritional benefits, poultry plays a vital role in the national economy as a revenue provider [1], through selling of eggs and meat [1,4,5]. Although poultry production specifically includes birds such as chickens, ducks, guineafowls, turkeys, pheasants, pigeons, quails, and ostriches domesticated for their meat and eggs [3,5,6,7], the production of chickens and turkeys contributes the most in commercial poultry farming [1,6,7].

Poultry production is affected by several diseases which are considered a main contributing factor to reduced production worldwide [3]. Previous studies have showed that several bacterial, viral, fungal, and parasitic infections result in significant economic losses, primarily associated with high morbidity and mortality rates, and increase management expenses [3,5,8]. However, parasitic infections rank high among these infections, and severely threaten poultry production [3,7,9,10]. Among several parasitic infections of poultry, haemoparasites are considered the most common [7], with unicellular eukaryotic parasites from the genera *Haemoproteus*, *Leucocytozoon*, *Plasmodium, Fallisia*, and *Trypanosoma* being the most reported [7,11,12,13] and well documented worldwide including Italy, Pakistan, Bolivia, Czechoslovakia, Kazakhstan, India, Tanzania, and Nigeria [4,14]. However, insufficient investigations have been conducted and documented on these parasitic faunas in sub-Saharan African countries [4], with most research focused on viral infections such as Newcastle disease, infectious bursal disease, fowl pox, avian influenza, and Marek’s disease among others [15], while haemoparasites have remained neglected [15]. As a result, global and regional knowledge and understanding of various haemoparasite species infecting poultry is still limited [11].

The life cycles of most of the haemoparasites of poultry are reported to be closely related [16]. Several studies have shown that the transmission of haemoparaistes is facilitated by various vectors, including black flies, lice, fleas, biting midges, and mosquitoes [14,16,17]. Infection of birds with haemoparasites mainly leads to anaemia, accompanied by many other severe pathologies depending on the species of the parasites and the organs affected [14,16,17]. However, the relationship between several haemoparasite species and their potential vectors has not been fully explored in several southern African countries; therefore, more research is still needed given the high mortalities and reduced productivity in poultry production, especially to resource-poor communities, due to haemoparasitic infections [7]. Furthermore, only a few studies have used or compared the morphological and advanced molecular techniques used in the detection and identification of these parasites [18,19,20,21] and consequently, the diversity of the parasites remains unknown, especially in the sub-Saharan Africa region [21,22]. Therefore, this review collated the existing records on the occurrence, distribution, and prevalence of haemoparasites of poultry in sub-Saharan Africa.

## 2. Materials and Methods

**Figure 1:**
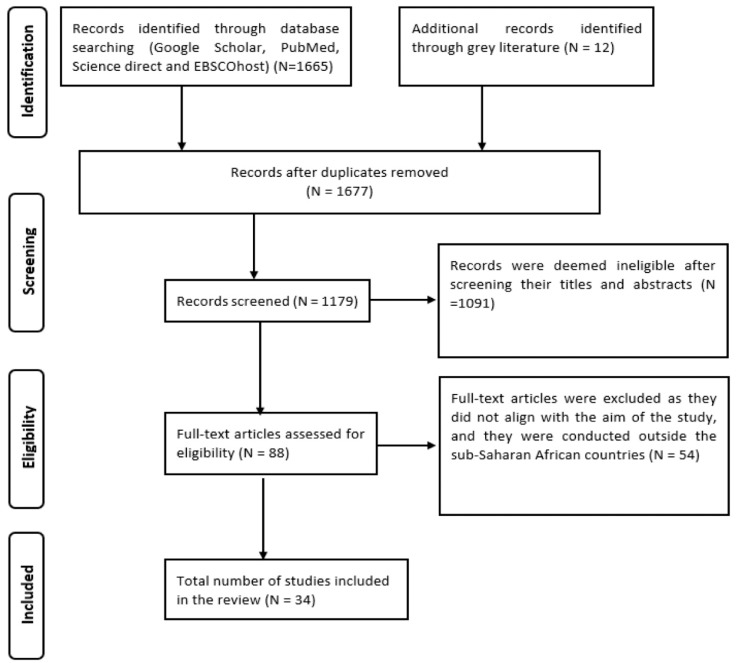
PRISMA diagram showing selection process.

The scoping review followed the guidelines and approach described by Arksey and O’Malley [23] in the following order: (i) identification of the scoping research question; (ii) searching for relevant articles; (iii) selection of relevant articles; (iv) charting of data; and (v) collating, summarizing, and reporting of results. The principles and guidelines for conducting and reporting a review were adopted from the ‘preferred reporting items for systematic reviews and meta-analysis’ (PRISMA) [24] (Figure 1).

### 2.1. Identification of the Research Question

The scoping review aimed to address the following questions: Which haemoparasites of poultry occur in sub-Saharan Africa? What is the distribution and prevalence of haemoparasite species infections in sub-Saharan Africa? Which poultry species are most susceptible to haemoparasites in sub-Saharan Africa? In order to address these questions, a comprehensive approach was adopted to search for peer-reviewed published articles explicitly reporting on the haemoparasites of poultry in sub-Saharan Africa. The procedure followed for the review process was consistent with the approach of a scoping review, i.e., to synthesize what is known about a particular matter across various literature forms in order to achieve clarity concerning the state of knowledge and evidence that exists [25].

### 2.2. Literature Search Strategy

A literature search was conducted on the Google Scholar, Science Direct, and PubMed databases using the following search terms and Boolean operators (AND, OR): distribution OR occurrence of haemoparasites, and prevalence of haemoparasites in poultry (chickens (*Gallus gallus domesticus*) OR ducks (*Anas platyrhyncos*) OR guinea fowls (*Numida meleagris*) OR turkeys (*Meleagris*) OR pheasants (*Phasianus colchicus*) OR pigeons (*Columba livia*) OR quails (*Coturnix coturnix)* OR ostriches (*Struthio camelus/molybdophanes*)) in sub-Saharan Africa (Angola OR Benin OR Botswana OR Burkina Faso OR Cameroon OR Cape Verde OR Central African Republic OR Chad OR Comoros OR Congo OR Côte d’lvoire OR Djibouti OR Equatorial Guinea OR Eritrea OR Ethiopia OR Gabon OR The Gambia OR Ghana OR Guinea OR Guinea-Bissau OR Kenya OR Lesotho OR Liberia OR Madagascar OR Malawi OR Mali OR Mauritania OR Mauritius OR Mozambique OR Namibia OR Niger OR Nigeria OR Réunion OR Rwanda OR Sao Tome and Principe OR Senegal OR Seychelles OR Sierra Leone OR Somalia OR South Africa OR Sudan OR Swaziland OR Tanzania OR Togo OR Uganda OR Western Sahara OR Zambia OR Zimbabwe); *Haemoproteus* OR *Leucocytozoon* OR *Plasmodium* OR *Trypanosoma* infections in poultry OR chickens OR ducks OR guinea fowls OR turkeys OR pheasants OR pigeons OR quails OR ostriches AND Sub-Saharan African countries.

The results from the search were screened by DT for inclusion through screening their abstracts and titles. Additionally, reference lists of the selected studies were screened to identify additional relevant articles that were not identified through the electronic database search. The full-text articles that were retrieved were managed in EndNote reference manager version x9 (Clarivate Analytics, Philadelphia, PA, USA).

### 2.3. Study Selection

Studies were included in the review if they were published in peer-reviewed journals between 1970 and 2021, and explicitly reported on (i) the occurrence of haemoparasites of poultry in sub-Saharan Africa countries, (ii) the distribution and prevalence of haemoparasites of poultry in sub-Saharan Africa countries, or (iii) the identification of haemoparasites of poultry from sub-Saharan Africa into genus and species levels. Articles were excluded from the review if (i) they were conducted outside the sub-Saharan African countries, (ii) they were published reviews and experimental studies, theses and books, and not written in English, or (iii) the information contained in the reviewed articles did not contribute to answering the scoping review questions. In cases where multiple studies were conducted in the same area, by the same authors, using the same hosts, and reported the same results, only one study was included in the review. The selection process followed is shown in Figure 1.

### 2.4. Charting the Data and Summarizing the Results

The following data were extracted from articles that met the above inclusion criteria: author name(s), year of publication, aim or objectives of the study, country where the study was conducted, type of study, sample size, species of parasites, diagnostic method(s) used for detection of haemoparasite, and outcomes of the study.

## 3. Results

### 3.1. Eligibility of Search Results

The literature search yielded 1665 articles from four electronic databases, comprising books, reports, reviews, dissertations/theses, abstracts, and duplicated articles (Figure 1). An additional twelve articles were obtained through bibliographic searches (snowballing). Four hundred and ninety-eight (*n* = 498) duplicated articles were removed, and one thousand and ninety-one (*n* = 1091) studies were deemed ineligible after screening their titles and abstracts. Eighty-eight (*n* = 88) full texts were retrieved and reviewed, and fifty-three (*n* = 55) studies were removed as they did not meet the eligibility criteria and/or contribute to answering the review questions. A total of thirty-three (*n* = 33) articles met the criteria and were included in this review (Figure 1).

Out of the 33 studies reviewed, 15 studies were conducted in Nigeria, 4 in Kenya, South Africa and Uganda had 3 studies each, Tanzania and Botswana had 2 studies each, Zimbabwe and Ghana had 1 study each, and 1 study was conducted in two countries (Cameroon and Uganda), (Zambia and Zimbabwe) (Table 1). Sixteen studies exclusively reported infections in chickens and seven in pigeons, two studies reported on guineafowls and ducks, and one on quails. Five studies assessed infections in more than one poultry species.

### 3.2. Occurrence and Geographical Distribution of Haemoparasites

The results show that 16 haemoparasite species from five (*n* = 5) genera *(Haemoproteus, Hepatazoon, Leucocytozoon, Plasmodium*, *and Trypanosoma)* of poultry have been documented in ten countries across sub-Saharan Africa (Table 1, Supplementary Table S1). Of these genera, *Leucocytozoon and Haemoproteus* were the most common and predominant genera, each reported in eight of ten countries where studies were conducted. Five *Leucocytozoon* species were documented, viz., *L. marchouxi, L. neavei, L. sabrazesi, L. schoutedeni*, and an *unidentified Leucocytozoon* spp. *Leucocytozoon marchouxi* was reported to infect pigeons in South Africa. *Leucocytozoon neavei* and *L. sabrazesi* infections were documented in one host only, with the former species found in guineafowls in South Africa, Zambia, and Zimbabwe, and the latter species found only in chickens from Zimbabwe. *Leucocytozoon schoutedeni* was reported in chickens from Cameroon, Kenya, Tanzania, and Uganda, and guineafowls from Tanzania. Infection by *Leucocytozoon* spp. was reported in multiple host species from Kenya (chickens, ducks), Nigeria (chickens, ducks, quails), and Uganda (chickens).

Three species from the *Haemoproteus* genus were identified, and this included *Haemoproteus columbae, Haemoproteus pratasi*, and *Haemproteus* spp*. Haemproteus* spp. infections were reported in Kenya, Nigeria, South Africa, and Uganda, infecting various poultry species ranging from chickens and ducks (Nigeria, Kenya, Uganda), pigeons (Nigeria, South Africa, Uganda), quails (Nigeria), guineafowls (Nigeria, Uganda), and turkeys (Uganda). *Haemoproteus columbae* occurred in Botswana, South Africa, and Tanzania, whilst *H. pratasi* was documented in South Africa, Zambia, and Zimbabwe, and these species infected pigeons and guineafowls, respectively. The results showed that four *Plasmodium* species*,* viz*., Plasmodium circumflexum, Plasmodium gallinaceum, Plasmodium juxtanucleare,* and *Plasmodium* spp. were identified across Ghana, Kenya, Nigeria, South Africa, Uganda, and Zimbabwe. *Plasmodium* spp. infections were identified in multiple poultry species from Kenya (chickens), Nigeria (chickens, guineafowls, ducks, turkeys, pigeons), and Uganda (chickens, ducks, turkeys, guineafowls, pigeons). *Plasmodium gallinaceum* was detected and identified in chickens from Kenya, Nigeria, and Zimbabwe, and quails from Nigeria. *Plasmodium circumflexum* and *P. juxtanucleare* were documented only in South African guineafowls and Ghanian chickens, respectively. Three *Typanosoma* species were documented in Zimbabwe, Uganda, and South Africa. *Trypanosoma avium* and *T. gallinarum* were reported to infect chickens in Zimbabwe and Uganda, while *T. numidae* was reported in guineafowls in South Africa.

### 3.3. Single Haemoparasite Species Infections

The prevalence results showed that chickens were the most studied and infected poultry species compared to other reported species (Table 2). The results further showed that within the *Leucocytozoon* genus*,* the lowest prevalence was recorded in Nigeria (0.8%, 1/125) [16] and the highest in Kenya (100%, 30/30) [26], both in chickens and based on microscopic examination. *Leucocytozoon schoutedeni* recorded the highest prevalence of 52.1% in chickens in Kenya based on microscopic examination [27], and only 5.57% in ducks in Nigeria [28], while the highest prevalence of *L. naevei* was 29% in guineafowls in Zimbabwe [29]. The results further showed the highest prevalence of *Haemoproteus* spp. in pigeons in South Africa (97%) based on both microscopy and molecular screening [30], and ducks showed a low prevalence of *Haemoproteus* spp.*,* with a prevalence of 0.8% in Nigeria based on microscopic examination [16]. Furthermore, *H. columbae*, only recorded in pigeons, showed a prevalence ranging from 11% in Tanzania [31] to 79.2% in Botswana [32] based on microscopic examination. The prevalence of *H. pratasi* ranged from 7 to 8% in guineafowls in Zimbabwe [29].

*Plasmodium* spp. infection in chickens recorded the highest prevalence of 100% (30/30) in Kenya [26] and the lowest (5%, 10/200) in Nigeria using microscopic examination [7]. In other poultry species, the lowest *Plasmodium* spp. infection was in ducks (0.68%) in Nigeria [28] and the highest in turkeys (40%) in Uganda [1] based on microscopic examination. *Plasmodium gallinaceum* infections in chickens ranged from 100% (13/13) in Nigeria to 53.7% (77/144) in Kenya based on microscopic examination. The prevalence of *Plasmodium juxtanucleare* in chickens was 27% (27/100) based on microscopic examination and was only reported in Ghana. *Plasmodium* spp. infections in pigeons ranged from 20% (30/150) in Nigeria [6] to 29% (10/34) in Uganda using microscopic examination [33].

The prevalence of infection of *Trypanosoma gallinarum* in chickens was only 7.8% (6/77) in Uganda based on microscopic examination and molecular screening [34].

### 3.4. Multiple Haemoparasite Species Infections

The results showed that mixed infections between two or more haemoparasites were common, and were reported in quails, pigeons, chickens, ducks, turkeys, and guineafowls, but predominantly in chickens (Table 3). The mixed infections recorded included triple infection of *Plasmodium* spp., *Leucocytozoon* spp., and *Haemoproteus* spp. in chickens in Kenya, with a prevalence of 38% (43/114) based on microscopic examinations. Co-infection of *Plasmodium* spp. and *Haemoproteus* spp. was reported in Uganda and Nigeria, with the prevalence ranging from 1.8% (37/2100) in chickens in Nigeria based on microscopic examination [35] to 71% (10/14) in turkeys in Uganda based on microscopic examination and molecular screening [36]. *Leucocytozoon schoutedeni* and *T.* g*allinarum* co-infection was reported in Uganda, with a prevalence of 2.6% (2/77) in chickens based on microscopic examination and molecular screening [34]. The results also showed that Nigeria recorded the majority of the mixed infections and these included *Plasmodium* spp. and *Leucocytozoon* spp., with the prevalence ranging from 0.9% (1/108) in chickens in Nigeria [37] to 7% (4/57) in quails in Nigeria [38], based on microscopic examination; *Haemoproteus* spp. and *Leucocytozoon* spp. in chickens, with a prevalence of 13% (14/108) in Nigeria based on microscopic examination [37]; and *Haemoproteus* spp. and *P. gallinaceum* in quails, with a prevalence of 9% (5/27) based on microscopic examination in Nigeria [38].

## 4. Discussion

The results from this study shows that the occurrence of haemoparasite species in poultry in sub-Saharan Africa has been documented in South Africa, Botswana, Zimbabwe, Zambia, Kenya, Tanzania, Uganda, Ghana, Nigeria, and Cameroon. According to Valkiūnas [39], avian haemasporidian parasites of the genera *Plasmodium*, *Haemoproteus*, *Parahaemoproteus*, and *Leucocytozoon* are considered as the most diverse group of vector-borne parasites, and can infect blood cells of a variety of avian species across all zoogeographical regions. This study recorded 16 species of avian haemoparasites from five genera. Amongst these, the genera *Plasmodium, Haemoproteus*, and *Leucocytozoon* were most common, and other genera such as *Hepatozoon* and *Trypanosoma* were also recorded. This was consistent with the report by Okanga et al. [40] which indicated that *Plasmodium*, *Haemoproteus*, and *Leucocytozoon* were the three common genera, together forming an umbrella of at least 206 species, with more than 4100 lineages infecting more than 9100 hosts, represented mostly by bird species [41].

Although reports of these parasites were predominantly documented in Nigeria, South Africa and Kenya reported a greater variety of species as compared to Nigeria. *Haemoproteus* and *Leucocytozoon* species infections were widespread and documented in eight of the ten countries where studies were carried out. The two genera were common in South Africa, Zimbabwe, Zambia, Kenya, Uganda, Tanzania, and Nigeria, however, *Haemoproteus* and *Leucocytozoon* were recorded as the only haemoparasites occurring in Botswana and in Cameroon, respectively. This was not surprising as *Haemoproteus* species are considered some of the most pathogenic haemoparasites of birds [42] and their infections have been reported in birds worldwide, except Antarctica [43].

The results showed that *Leucocytozoon* and *Plasmodium* recorded the highest number of species as compared to other genera. *Leucocytozoon* recorded the highest number of species, and these included *L. marchouxi, L. neavei, L. sabrazesi, L. schoutedeni*, and unidentified *Leucocytozoon* spp. According to Win et al. [44], there are currently over 100 species of *Leucocytozoon* globally, however, only a few have been documented in poultry and these include *L. caulleryi*, L*. sabrazesi*, and *L. schoutedeni* which are common in chickens, and *L. smithi* in turkeys and *L. simondi* in waterfowls [44]. However, only two of these species (*L. sabrazesi* and *L. schoutedeni)* were reported in chickens in the reviewed studies. Of the commonly listed *Leucocytozoon* species in chickens, several authors regarded *L. caulleryi* as the most pathogenic species in chickens [44,45]. Although this species was not documented in the reviewed studies in sub-Saharan Africa, other species documented in this review have been found in other countries and localities such as Myanmar, southeast Asia [44].

The aetiology of avian malaria, *Plasmodium* spp., recorded the second highest number of species in sub-Saharan Africa. According to Valkiūnas and Iezhova [46], morphological and DNA sequence data indicated the presence of 55 distinguishable avian *Plasmodium* species. These species are common and widespread globally except Antarctica, but their pathogenic effects have been inadequately studied in both wild and domestic birds [47]. The reviewed studies reported the presence of *P. circumflexum, P. gallinaceum, P. juxtanucleare*, and *Plasmodium* spp. in sub-Saharan Africa. *Plasmodium gallinaceum*, *P. juxtanucleare*, and *P. durae* have been shown to be the most pathogenic for poultry, causing up to 90% mortality [47], and in chickens, *P. gallinaceum* and *P. juxtanucleare* are well-known as being pathogenic [48].

The results showed that the majority of the studies employed solely microscopic examination to detect haemoparasitic infection, hence the observed lower resolution to species level in most studies. Several authors consider this diagnostic method as the traditional method and gold standard to diagnose *Plasmodium* infection in both humans and birds [40,48]. Parasites are identified on a stained blood smear and identified based on the morphological features [11,49]. However, studies have also shown that PCR amplification and sequencing of the *cyt b* gene has contributed to the discovery of unique lineages, resulting in a large database of avian haemosporidian parasites (MalAvi) for comparison of sequences. Furthermore, the *cyt b* sequences also provided new opportunities for studying the host range, geographical distribution, ecology, genetic diversity and evolution of the haemoparasites [50,51,52]. Although this technique alone has enabled the detection of very light parasitemia of haemosporidian parasites [53], several authors have previously warned against biased amplification, which often underestimates the number of species present in cases where mixed infections occur [54,55,56]. Hence, continued use of morphological/microscopic examination as a complementary technique, as shown in some studies, is recommended.

The reviewed studies showed that domesticated chickens had the highest prevalence (100%) of *Plasmodium* spp. and *Leucocytozoon* spp. infection in Kenya [26], based on microscopic examination. The reported prevalence of *Plasmodium* spp. and *Leucocytozoon* spp. infections are similar to the prevalence of 100% reported in Brazil [57] and 86.8–100% reported in areas surrounding Myanmar, in Asia [44]. The prevalence of *Leucocytozoon* spp. infection reported in Kenya was noted to be higher than the reported prevalence in Thailand [58,59], Myanmar [44], Colombia [60], Iran [61], and California [34]. The highest prevalence of *Haemoproteus* spp. was documented in ducks in Nigeria. Surprisingly, the lowest prevalence of *Leucocytozoon* spp. was in the same species (chickens) from Nigeria. According to Fecchio et al. [62], haemosporidian parasites demonstrate a wide variation in prevalence, however, the drivers of this variation across zoogeographical realms is only partially understood from region-level studies [62]. The lowest prevalence of *Plasmodium* spp. and *Haemoproteus* spp. was reported in different species, i.e., ducks and chickens, respectively, and a similar pattern was observed with other haemoparasites. The variation in prevalence of haemoparasites in avian species may be due to differences in the susceptibility of hosts, the presence of potential vectors, the differences in species or strain of the vectors and the possibility of the exposure of hosts to vectors, geographical and climatic conditions which affect the distribution and spread of vectors, and the avian health management programme, including vector prevention and control strategy, in a given country [7,63,64,65,66].

The results showed the presence of mixed infections of two or more haemoparasites in poultry but predominantly in chickens. Co-infection by *Plasmodium* spp. and *Haemoproteus* spp. were the most common in chickens from Uganda and Nigeria. According to Lawal et al. [7] mixed infection of the combination of these two haemoparasites has been previously reported in village chickens from various developing countries, however, the prevalence of infection varied with localities. Furthermore, several authors supported that mixed infection by these haemoparasite taxa are not only the most commonly reported in poultry mainly in village/scavenging chickens, but they are also distributed throughout the world [35,36]. According to Lawal et al. [35], the prevalence of this mixed infection may be due to the geographical distribution of these species, coupled with the availability of vectors in areas from which they were documented. Mixed infection by *Leucocytozoon schoutedeni* and *Trypanosoma gallinarum* species, reported in chickens with a prevalence of 2.6% (2/77), was the least common and reported only in Uganda [34]. According to Sabuni et al. [27], this mixed infection may have been due to changes in climatic conditions which affected the adaptation of *L. schoutedeni* and *T. gallinarum* and the distribution of their vectors. The results also recorded the presence of mixed infection by three haemoparasite species (*Plasmodium* spp., *Leucocytozoon* spp., and *Haemoproteus* spp.) in chickens from Kenya, with a prevalence of 38%. Similar triple mixed infection was previously reported in pigeons (2.5%) from Bangladesh [67]. According to Sadiq et al. [68], the occurrence of this triple infection in chickens may be associated with the environmental factors that stimulate the survival and existence of the vectors and the exposure of the host species.

## 5. Conclusions

The results from this review recorded 16 haemoparasites in six poultry species across eight sub-Saharan African countries. Infections due to *Haemoproteus* and *Leucocytozoon* species were the most common, and documented in all eight countries. Most studies and infections were documented in chickens followed by pigeons. The highest prevalence of infections was recorded in chickens (100%) by *Plasmodium* spp., *Plasmodium gallinaceum*, and *Leucocytozoon* spp. The results also showed that most studies used solely morphological/microscopic examination to detect infection and identify species, with only a few using a combination of both microscopy and molecular/PCR-based techniques. However, the use of microscopic techniques only can easily lead to misidentification, or the inability to distinguish between species of the same genus, as observed in some of the reviewed studies. Therefore, we recommend the use of PCR to distinguish between species and study the genetic diversity and evolution of the haemoparasites of poultry, complemented by microscopic examination to increase the chances of detecting infections by multiple species. Future studies could focus not only on the infections in poultry, but encompass the identity and assessment of the abundance of vectors, especially in areas with high infection rates. Furthermore, studies should be conducted to assess the impact of climate change on the vectors and infections in poultry. Moreover, the economic impact of haemoparasite infection in poultry should be critically researched to provide more knowledge and awareness on the economic loss experienced by substantial, small-scale, and commercial farmers as a result of haemoparasite infections. This information is useful in improving existing and developing new preventive and probe control measures of haemoparasites of poultry by the combined efforts of researchers/epidemiologists, farmers, and the government.

## Figures and Tables

**Table 1:** Checklist of poultry haemoparasite species reported in sub-Saharan Africa between 1970 and 2021.

Haemoparasite Species	Country of Study	Host Studied	Author and Year
*Haemoproteus columbae*	Botswana, South Africa, Tanzania	Pigeons	Msoffe et al., 2010; Mushi et al., 2000; Mushi et al., 1999; Earle, 1993b
*Haemoproteus pratasi*	South Africa, Zambia, Zimbabwe	Guineafowls	Earle, 1993a; Earle et al., 1991
*Haemproteus* spp.	Kenya, Nigeria, South Africa, Uganda	Chickens, ducks, pigeons, guineafowls, quails, turkey	Jubril et al., 2021; Lawal et al., 2021a; Lawal et al., 2021b; Lawal et al., 2021c; Nebel et al., 2020; Wamboi et al., 2020; Idowu et al., 2019; Lawal et al., 2019; Nakayima et al., 2019; Waruiru et al., 2017; Ogbaje et al., 2019; Mohammed et al., 2019; Lawal et al., 2016; Opara et al., 2012; Sabuni et al., 2011; Dranzoa et al., 1999;
*Hepatozoon* spp.	South Africa	Guineafowls	Earle et al., 1991
*Leucocytozoon marchouxi*	South Africa	Pigeons	Earle, 1993b
*Leucocytozoon neavei*	South Africa, Zambia, Zimbabwe	Guineafowls	Earle, 1993a; Earle et al., 1991
*Leucocytozoon sabrazesi*	Zimbabwe	Chickens	Permin et al., 2002
*Leucocytozoon schoutedeni*	Cameroon, Kenya, Tanzania, Uganda	Chickens, guineafowls	Chege et al., 2015; Sabuni et al., 2011; Sehgal et al., 2006; Fallis et al., 1973
*Leucocytozoon* spp.	Kenya, Nigeria, Uganda	Chickens, ducks, quails, guineafowls	Jubril et al., 2021; Lawal et al., 2021b; Wamboi et al., 2020; Idowu et al., 2019; Lawal et al., 2019; Mohammed et al., 2019; Nakayima et al., 2019; Ogbaje et al., 2019; Waruiru et al., 2017; Opara et al., 2014
*Plasmodium circumflexum*	South Africa	Guineafowls	Earle et al., 1991
*Plasmodium gallinaceum*	Kenya, Nigeria, Zimbabwe	Chickens, quails	Jubril et al., 2021; Chege et al., 2015; Sam-Wobo et al., 2014; Usman et al., 2012; Sabuni et al., 2011; Permin et al., 2002
*Plasmodium juxtanucleare*	Ghana	Chickens	Poulsen et al., 2000
*Plasmodium* spp.	Kenya, Nigeria, Uganda	Chickens, guineafowls, pigeons, ducks, turkey	Lawal et al., 2021a; Lawal et al., 2021b; Lawal et al., 2021c; Wamboi et al., 2020; Idowu et al., 2019; Lawal et al., 2019; Ogbaje et al., 2019; Mohammed et al., 2019; Nakayima et al., 2019; Lawal et al., 2016; Maxwell et al., 2016; Opara et al., 2012; Opara et al., 2014; Igbokwe et al., 2008; Dranzoa et al., 1999; Illango et al., 2002
*Trypanosoma avium*	Zimbabwe	Chickens	Permin et al., 2002
*Trypanosoma gallinarum*	Uganda	Chickens	Sehgal et al., 2006
*Trypanosoma numidae*	South Africa	Guineafowls	Earle et al., 1991

**Table 2:** Prevalence of haemoparasite single species infections in poultry from sub-Saharan Africa between 1970 and 2021.

Country of Study	Haemoparasite Species	Host Studied	Total Examined	Total Positive	Prevalence (%)	Diagnostic Method	Target Gene/Primer	Author and Year
Botswana	*Haemopr * * oteus * * columbae *	Pigeons	24	19	79.2	Microscopic examination		Mushi et al., 2000
Cameroon	*Leucocytozoon schoutedeni*	Chickens	71	5	7	Microscopic examination and PCR	Cyt B (HaemFl and HaemR2L)	Sehgal et al., 2006
Ghana	*Plasmodium juxtanucleare*	Chickens	100	27	27	Microscopic examination		Poulsen et al., 2000
Kenya	*Leucocytozoon* spp.	Chickens	30	30	100	Microscopic examination		Wamboi et al., 2020
Kenya	*Haemoproteus* spp.	Chickens	30	9	30	Microscopic examination		Wamboi et al., 2020
Kenya	*Plasmodium* spp.	Chickens	30	30	100	Microscopic examination		Wamboi et al., 2020
Kenya	*Plasmodium gallinaceum*	Chickens	144	77	53.7	Microscopic examination		Sabuni et al., 2011
Kenya	*Leucocytozoon schoutedeni*	Chickens	144	75	52.1	Microscopic examination		Sabuni et al., 2011
Kenya	*Haemoproteus* spp.	Chickens	144	5	3.5	Microscopic examination		Sabuni et al., 2011
Kenya	*Plasmodium gallinaceum*	Chickens	24 24	19 15	79.2 * 62.5 ^#^	Microscopic examination		Chege et al., 2015
Kenya	*Leucocytozoon schoutedeni*	Chickens	24 24	7 3	29.2 * 12.5 ^#^	Microscopic examination		Chege et al., 2015
Kenya	*Leucocytozoon* spp.	Ducks	145	10	6.9	Microscopic examination		Waruiru et al., 2017
Kenya	*Haemoproteus* spp.	Ducks	145	5	3.5	Microscopic examination		Waruiru et al., 2017
Nigeria	*Haemoproteus* spp.	Chickens	2100	55	2.6	Microscopic examination		Lawal et al., 2021a
Nigeria	*Plasmodium* spp.	Chickens	2100	198	9.4	Microscopic examination		Lawal et al., 2021a
Nigeria	*Plasmodium gallinaceum*	Chickens	100	13	13	Microscopic examination		Sam-Wobo et al., 2014
Nigeria	*Plasmodium* spp.	Chickens	530	69	13	Microscopic examination		Lawal et al., 2021b
Nigeria	*Haemoproteus* spp.	chickens	530	27	5.1	Microscopic examination		Lawal et al., 2021b
Nigeria	*Leucocytozoon* spp.	Chickens	530	4	0.8	Microscopic examination		Lawal et al., 2021b
Nigeria	*Plasmodium* spp.	Chickens	500	150	30	Microscopic examination		Maxwell et al., 2016
Nigeria	*Plasmodium* spp.	Chickens	220	49	22.27	Microscopic examination		Ogbaje et al., 2019
Nigeria	*Haemoproteus* spp.	Chickens	125	1	0.8	Microscopic examination		Ogbaje et al., 2019
Nigeria	*Leucocytozoon* spp.	Chickens	125	1	0.8	Microscopic examination		Ogbaje et al., 2019
Nigeria	*Plasmodium* spp.	Chickens	346	41	11.8	Microscopic examination		Lawal et al., 2021c
Nigeria	*Haemoproteus* spp.	Chickens	346	23	6.8	Microscopic examination		Lawal et al., 2021c
Nigeria	*Plasmodium* spp.	Chickens	108	59	54.6	Microscopic examination		Mohammed et al., 2019
Nigeria	*Haemoproteus* spp.	Chickens	108	1	0.9	Microscopic examination		Mohammed et al., 2019
Nigeria	*Leucocytozoon* spp.	Chickens	108	1	0.9	Microscopic examination		Mohammed et al., 2019
Nigeria	*Plasmodium* spp.	Chickens	425	41	9.6	Microscopic examination		Igbokwe et al., 2008
Nigeria	*Plasmodium* spp.	Guineafowls	150	13	8.7	Microscopic examination		Igbokwe et al., 2008
Nigeria	*Haemoproteus* spp.	Pigeons	150	90	60	Microscopic examination		Opara et al., 2012
Nigeria	*Plasmodium* spp.	Pigeons	150	30	20	Microscopic examination		Opara et al., 2012
Nigeria	*Leucocytozoon* spp.	Ducks	880	49	5.57	Microscopic examination		Lawal et al., 2019
Nigeria	*Plasmodium* spp.	Ducks	880	6	0.68	Microscopic examination		Lawal et al., 2019
Nigeria	*Haemoproteus* spp.	Ducks	880	19	2.16	Microscopic examination		Lawal et al., 2019
Nigeria	*Haemoproteus* spp.	Chickens	200	18	9	Microscopic examination		Lawal et al., 2016
Nigeria	*Plasmodium* spp.	Chickens	200	10	5	Microscopic examination		Lawal et al., 2016
Nigeria	*Plasmodium gallinaceum*	Chickens	100	12	12	Microscopic examination		Usman et al., 2012
Nigeria	*Plasmodium* spp.	Chickens	60	14	23	Microscopic examination		Idowu et al., 2019
Nigeria	*Leucocytozoon* spp.	Chickens	60	4	7	Microscopic examination		Idowu et al., 2019
Nigeria	*Haemoproteus* spp.	Chickens	60	2	3	Microscopic examination		Idowu et al., 2019
Nigeria	*Plasmodium* spp.	Guineafowls	60	9	15	Microscopic examination		Idowu et al., 2019
Nigeria	*Haemoproteus* spp.	Guineafowls	60	2	3.3	Microscopic examination		Idowu et al., 2019
Nigeria	*Haemoproteus* spp.	Quails	57	18	32	Microscopic examination		Jubril et al., 2021
Nigeria	*Plasmodium gallinaceum*	Quails	57	6	11	Microscopic examination		Jubril et al., 2021
Nigeria	*Leucocytozoon* spp.	Quails	57	5	9	Microscopic examination		Jubril et al., 2021
Nigeria	*Leucocytozoon* spp.	Quails	5040	448	9	Microscopic examination		Opara et al., 2014
Nigeria	*Plasmodium* spp.	Turkeys	560	224	40	Microscopic examination		Opara et al., 2014
Zambia	* Haemoproteus pratasi *	Guineafowls	20	3	15	Microscopic examination		Earle et al., 1993a
Zambia	*Leucocytozoon * * neavei *	Guineafowls	20	4	20	Microscopic examination		Earle et al., 1993a
Zimbabwe	* Haemoproteus pratasi *	Guineafowls	14	1	7	Microscopic examination		Earle et al., 1993a
Zimbabwe	*Leucocytozoon * * neavei *	Guineafowls	14	4	29	Microscopic examination		Earle et al., 1993a
Zimbabwe	* Haemoproteus pratasi *	Guineafowls	12	1	8	Microscopic examination		Earle et al., 1993a
South Africa	* Haemoproteus columbae *	Pigeons	50	6	12	Microscopic examination		Earle, 1993b
South Africa	* Haemoproteus columbae *	Pigeons	33	24	73	Microscopic examination		Earle, 1993b
South Africa	* Haemoproteus * spp.	Pigeons	192	186	97	Microscopic examination and molecular screening	*cyt b* (HPL-intF1 and HPL-intR1)	Nebel et al., 2020
Tanzania	*Haemopr * * oteus * * columbae *	Pigeons	100	11	11	Microscopic examination		Msoffe et al., 2010
Tanzania	*Haemopr * * oteus * * columbae *	Pigeons	100	63	63	Microscopic examination		Msoffe et al., 2010
Uganda	*Haemoproteus * spp.	Pigeons	34	26	77	Microscopic examination		Dranzoa et al., 1999
Uganda	*Plasmodium* spp.	Pigeons	34	10	29	Microscopic examination		Dranzoa et al., 1999
Uganda	*Haemoproteus * spp.	Chickens	304	10	3	Microscopic examination and molecular screening	*cyt b* (DW2 and DW4, LCytb-F and LCytb-R)	Nakayima et al., 2019
Uganda	*Plasmodium* spp.	Chickens	304	66	22	Microscopic examination and molecular screening	*cyt b* (DW2 and DW4, APF and APRN)	Nakayima et al., 2019
Uganda	*Leucocytozoon * spp.	Chickens	304	7	2	Microscopic examination and molecular screening	*cyt b* (DW2 and DW4, LCytb-F and LCytb-R)	Nakayima et al., 2019
Uganda	*Haemoproteus * spp.	Ducks	70	44	63	Microscopic examination and molecular screening	*cyt b* (DW2 and DW4, L15183 and H15730)	Nakayima et al., 2019
Uganda	*Plasmodium* spp.	Ducks	70	18	26	Microscopic examination and molecular screening	*cyt b* (DW2 and DW4, APF and APRN)	Nakayima et al., 2019
Uganda	*Haemoproteus * spp.	Turkeys	14	7	50	Microscopic examination and molecular screening	*cyt b* (DW2 and DW4, L15183 and H15730)	Nakayima et al., 2019
Uganda	*Plasmodium* spp.	Turkeys	14	3	21	Microscopic examination and molecular screening	*cyt b* (DW2 and DW4, APF and APRN)	Nakayima et al., 2019
Uganda	*Haemoproteus * spp.	Guineafowls	19	8	42	Microscopic examination and molecular screening	*cyt b* (DW2 and DW4, L15183 and H15730)	Nakayima et al., 2019
Uganda	*Plasmodium* spp.	Guineafowls	19	1	5	Microscopic examination and molecular screening	*cyt b* (DW2 and DW4, APF and APRN)	Nakayima et al., 2019
Uganda	*Leucocytozoon schoutedeni *	Chickens	77	22	28.6	Microscopy and PCR-based methods	*cyt b* (HaemFL and HaemR2L)	Sehgal et al., 2006
Uganda	*Trypanosoma gallinarum *	Chickens	77	6	7.8	Microscopy and PCR-based methods	SSU rRNA (S-1755 and S-823)	Sehgal et al., 2006

* Wet season; ^#^ dry season.

**Table 3:** Prevalence of mixed haemoparasite infections in poultry from sub-Saharan Africa between 1970 and 2021.

Country of Study	Haemoparasite Species	Host Studied	Total Examined	Total Positive	Prevalence (%)	Diagnostic Method	Target Gene/Primers	Author and Year
Kenya	*Plasmodium* spp., *Leucocytozoon* spp., and *Haemoproteus* spp.	Chickens	114	43	38	Microscopic examination		Sabuni et al., 2011
Nigeria	*Plasmodium* spp., and *Haemoproteus* spp.	Chickens	2100	37	1.8	Microscopic examination		Lawal et al., 2021a
Nigeria	*Plasmodium* spp., and *Haemoproteus* spp.	Chickens	530	26	4.9	Microscopic examination		Lawal et al., 2021b
Nigeria	*Plasmodium* spp. and *Haemoproteus* spp.	Chickens	346	8	2.3	Microscopic examination		Lawal et al., 2021c
Nigeria	*Plasmodium* spp. and *Leucocytozoon* spp.	Chickens	108	14	13	Microscopic examination		Mohammed et al., 2019
Nigeria	*Haemoproteus* spp. and *Leucocytozoon* spp.	Chickens	108	1	0.9	Microscopic examination		Mohammed et al., 2019
Nigeria	*Plasmodium* spp. and *Haemoproteus* spp.	Chickens	108	5	4.6	Microscopic examination		Mohammed et al., 2019
Nigeria	*Plasmodium* spp. and *Haemoproteus* spp.	Chickens	200	6	3	Microscopic examination		Lawal et al., 2016
Nigeria	*Haemoproteus* spp. and *Plasmodium gallinaceum*	Negative Japanese quails	57	5	9	Microscopic examination		Jubril et al., 2021
Nigeria	*Haemoproteus* spp. and *Leucocytozoon* spp.	Negative Japanese quails	57	4	7	Microscopic examination		Jubril et al., 2021
Uganda	*Plasmodium* spp. and *Haemoproteus* spp.	Pigeons	34	10	29	Microscopic examination		Dranzoa et al., 1999
Uganda	*Plasmodium* spp. and *Haemoproteus* spp.	Chickens	304	81	27	Microscopic examination and molecular screening	*cyt b* (DW2 and DW4, APF and APRN, L15183 and H15730)	Nakayima et al., 2019
Uganda	*Plasmodium* spp. and *Haemoproteus* spp.	Ducks	70	43	61	Microscopic examination and molecular screening	*cyt b* (DW2 and DW4, APF and APRN, L15183 and H15730)	Nakayima et al., 2019
Uganda	*Plasmodium* spp. and *Haemoproteus* spp.	Turkeys	14	10	71	Microscopic examination and molecular screening	*cyt b* (DW2 and DW4, APF and APRN, L15183 and H15730)	Nakayima et al., 2019
Uganda	*Plasmodium* spp. and *Haemoproteus* spp.	Guineafowls	19	9	47	Microscopic examination and molecular screening	*cyt b* (DW2 and DW4, APF and APRN, L15183 and H15730)	Nakayima et al., 2019
Uganda	*Leucocytozoon schoutedeni* and *Trypanosoma gallinarum*	Chickens	77	2	2.6	Microscopy and PCR-based methods	*cyt b* (HaemFL and HaemR2L, S-755 and S-823)	Sehgal et al., 2006

## Data Availability

Not applicable.

## Supplementary Materials

The following supporting information can be downloaded at: https://www.mdpi.com/article/10.3390/pathogens12070945/s1, Table S1: Summary of studies reporting occurrence and detection of haemoparasites in poultry in sub-Saharan Africa between 1970 and 2021.
