# Supplementary material for: Occurrence, Prevalence, and Distribution of Haemoparasites of Poultry in Sub-Saharan Africa: A Scoping Review
Source: Pathogens. 2023 Jul 17;12(7):945. doi: 10.3390/pathogens12070945 (PMC10386234; doi:10.3390/pathogens12070945)
Supplement: Supplementary file 1 [file pathogens-12-00945-s001.zip › pathogens-2486887-supplementary.pdf]

**Supplementary Table S1.** Summary of studies reporting occurrence and detection of haemoparasites in poultry in sub-Saharan Africa between 1970-2021.

| Reference | Objective (s) of study | Host studied | Haemoparasite species reported | Outcomes of the study | Country of study |
|---|---|---|---|---|---|
| Jubril et al., 2021 | To evaluate the incidence of haemoparasites in commercial quails and their associated haematology changes. | Quails | *Haemoproteus* spp., *Plasmodium gallinaceum, Leucocytozoon* spp. | - *Haemoproteus* spp., *Plasmodium gallinaceum, Leucocytozoon* spp. were identified<br>- *Haemoproteus* spp. was the most abundant, followed by *Leucocytozoon* spp.<br>- 29 (51 %) quails were positive for single infection while 9 (16 %) were positive for mixed infection<br>- The results showed that there is a high occurrence of haemoparasitic infection in apparently healthy quails | Nigeria |
| Lawal et al., 2021a | To determine the emerging avian haemosporidian infections in Village chickens in Yobe State, Nigeria using microscopy. | Chickens | *Plasmodium* spp., *Haemoproteus* spp. | - *Plasmodium* spp. was the most commonly detected blood parasite infecting village chickens<br>- Mixed infection by *Plasmodium* spp. and *Haemoproteus* spp. was detected in chickens.<br>- A higher prevalence was observed in males (9.9 %) compared to female (4.0 %) village chickens.<br>- Avian haemosporidian infections were higher in adult chickens (10.4 %) than in the growers (3.4 %) | Nigeria |
| Lawal et al., 2021b | To determine the genera of haemoparasites that causes avian malaria in village chickens in Gombe Local Government Area of Gombe State, Nigeria. | Chickens | *Plasmodium* spp., *Haemoproteus* spp., *Leucocytozoon* spp. | - *Plasmodium* spp., *Haemoproteus* spp. and *Leucocytozoon* spp. were detected in single or mixed infections<br>- *Plasmodium* spp. was the most abundant parasite infecting chickens, followed by *Haemopr*oteus spp. and *Leucocytozoon* spp. was the least parasite detected<br>-Mixed infections were observed between *Plasmodium* spp. and *Haemoproteus* spp.<br>-The prevalence rates of reported haemoparasites were higher during the rainy season as compared to the dry season | Nigeria |
| Lawal et al., 2021c | To determine the prevalence of avian haemosporidian parasites in village chickens in Kwami, Gombe State, Nigeria. | Chickens | *Plasmodium* spp., *Haemoproteus* spp. | - *Plasmodium* was the most prevalent species (11.8 %) compared to *Haemoproteus* species (6.6 %)<br>- Prevalence of avian haemosporidian infections were higher in males (13.9 %) as compared to hens (6.9 %), also in adults (14.5 %) than growers (6.4 %)<br>- The prevalence of infection was higher during the rainy season (15.6 %) compared to the dry (5.2 %) | Nigeria |

| | | | | | |
|---|---|---|---|---|---|
| Nebel et al., 2020 | To assess blood parasite diversity by PCR screening for *H. columbae* lineages and by sequencing a section of the mitochondrial cyt b gene. | Pigeons | *Haemoproteus* spp. | - *Haemoproteus* spp. was the only detected species infecting pigeons<br>- High number of pigeons were infected with more than one *Haemoproteus* parasite lineage<br>- 6 of 192 all examined blood smears did not show any infection with haemoparasites based on the blood smear screening, however, four of these samples tested positive through molecular analysis. | South Africa |
| Wamboi et al., 2020 | To determine parasitic prevalence and probable haemato-biochemical changes that may occur from parasitic infections in marketed indigenous chickens in Kiambu county, Kenya. | Chickens | *Leucocytozoon* spp., *Haemoproteus* spp., *Plasmodium* spp. | - *Leucocytozoon* spp. and *Plasmodium* spp. infections were found in all chickens examined<br>- *Leucocytozoon* spp., *Haemoproteus* spp. and *Plasmodium* spp. were found as three mixed infections<br>- *Haemoproteus* spp. was the least encountered parasite in chickens studied | Kenya |
| Idowu et al., 2019 | To determine the prevalence and intensity of *Eimeria* spp. and haemosporidian infections of domestic chickens and helmeted guinea fowls sold at selected LBMs in Lagos State. | Chickens and Guinea fowls | *Plasmodium* spp., *Leucocytozoon* spp., *Haemoproteus* spp. | - Domestic chickens were infected by *Plasmodium* spp., *Leucocytozoon* spp. and *Haemoproteus* spp.<br>- Guinea fowls were only infected by *Plasmodium* spp. and *Haemoproteus* spp.<br>- More *Plasmodium* spp. infection were observed in male guinea fowls (26 %) compared to female (6 %) guinea fowls | Nigeria |
| Lawal et al., 2019 | To detect haemosporidians from the blood of Muscovy ducks in Gombe State Nigeria. | Ducks | *Leucocytozoon* spp., *Haemoproteus* spp., *Plasmodium* spp. | - *Leucocytozoon* spp. was the most abundant (5.57 %) haemoparasite, followed by *Haemoproteus* spp. (2.16 %) and *Plasmodium* spp. (0.68 %) were the least detected.<br>- Female ducks showed high prevalence of haemoparasites (18.54 %) compared to male ducks (13.58%)<br>- The highest prevalence infections were observed in the rainy season (24.55 %) than in the dry season (7.27 %) | Nigeria |
| Mohammed et al., 2019 | To determine the haemo- and endoparasites of local chickens reared in Gwagwalada Area Council, Abuja. | Chickens | *Plasmodium* spp., *Leucocytozoon* spp., *Haemoproteus* spp. | - *Plasmodium* spp. was the most abundant (54.6%), followed by both *Leucocytozoon* spp. and *Haemoproteus* spp (0.9 %)<br>- The occurrence of haemoparasites infections were higher in females (53.1 %) compared to males (46.9 %) | Nigeria |
| Nakayima et al., 2019 | To investigate the distribution, prevalence and diversity of haemosporidian parasites in free-ranging domestic birds in Uganda, East Africa | Chickens, ducks, turkeys, guinea fowls | *Haemoproteus* spp., *Plasmodium* spp., *Leucocytozoon* spp. | - Chickens were infected by *Haemoproteus* spp., *Plasmodium* spp., *Leucocytozoon* spp.<br>- *Plasmodium spp.* was the most abundant, followed by *Haemoproteus* spp., and *Leucocytozoon* spp. was the least detected in chickens. | Uganda |

| | | | | | |
|---|---|---|---|---|---|
| | | | | - Ducks, turkeys, and guinea fowls were only infected with *Haemoproteus* spp. and *Plasmodium* spp.<br>- Mixed infections of *Haemoproteus* spp. and *Plasmodium* spp. was detected in chicken, ducks, turkey, guinea fowls | |
| Ogbaje et al., 2019 | To investigate the occurrence, prevalence and some possible risk factors in the study area to sensitize and provide some guidance to the experts in designing effective control programme against those parasitic infections for poultry farmers in the area | Chickens | *Plasmodium* spp., *Haemoproteus* spp., *Leucocytozoon* spp. | - Only *Plasmodium* spp., *Haemoproteus* spp. and *Leucocytozoon* spp. were found in the chickens<br>- *Plasmodium* spp. was the most commonly detected species in broiler chickens (21.6 %), followed by *Haemoproteus* spp. (0.8 %) and *Leucocytozoon* spp. (0.8 %)<br>- The prevalence was higher in local chickens (23.16 %) compared to that of broiler chickens (21.60 %)<br>- The infections were more in male chickens (27.2 %) than in female chickens (26.1 %) | Nigeria |
| Waruiru et al., 2017 | To determine the prevalence of endo- and haemoparasites in different age and sex groups of free-range local ducks in Nairobi and its environs. | Ducks | *Leucocytozoon* spp., *Haemoproteus* spp. | - Blood smear revealed the presence of only two haemoparasites (*Leucocytozoon* spp., *Haemoproteus* spp.) in ducks | Kenya |
| Lawal et al., 2016 | To determine the prevalence and type of avian haemoparasites parasitizing village chickens in Maiduguri, Northeastern Nigeria and to provide data that can serve as reference in future research on village chickens in the study area. | Chickens | *Haemoproteus* spp., *Plasmodium* spp. | - *Haemoproteus* spp. and *Plasmodium* spp. were the only haemoparasites detected from the examined chickens<br>- *Haemoproteus* spp. was the most abundant compared to the *Plasmodium* spp.<br>- 17 % (34/200) chickens were positive for haemoparasite infections<br>- Mixed infections by two species was detected in village chickens | Nigeria |
| Maxwell et al., 2016 | To investigate the prevalence of haemoparasites of chickens in Owerri, southeastern Nigeria and to validate the scanty studies carried out in this part of the country. | Chickens | *Plasmodium* spp. | - *Plasmodium* infections were more in exotic chickens (66.7 %) compared to local chickens (33.3 %)<br>- *Plasmodium* spp. infections in exotic chickens were more in cocks (62.5 %) compared to hens (37.5 %)<br>- In the infected local chickens, the hens were more infected (85.7 %) than the cocks (14.3 %). | Nigeria |
| Chege et al., 2015 | To establish parasite types and intensity in village chicken of Mbeere Sub-County in dry and wet seasons. | Chickens | *Plasmodium gallinaceum, Leucocytozoon schoutedeni* | - *Plasmodium gallinaceum* was the most common haemoparasite detected<br>- The incident rates of were more during the wet season (79.2 %, 19/24) compared to the dry season (62.5 %, 15/24) | Kenya |

| | | | | | |
|---|---|---|---|---|---|
| Opara et al., 2014 | - | Chickens and turkeys | *Leucocytozoon* spp., *Plasmodium* spp. | - *Leucocytozoon* spp. was the only blood parasites found in chickens with a prevalence rate of 448 (8.9 %) occurring in both male and female chickens<br>- Only *Plasmodium* sp. was found in the turkeys with a prevalence of 224 (40 %)<br>- Blood infection was higher in the male chickens (18.2 %) as compared to the female chickens (5.9 %) | Nigeria |
| Sam-Wobo et al., 2014 | To determine malaria parasites and packed cell volume on free range poultry in Abeokuta. | Chickens | *Plasmodium gallinaceum* | - Only *Plasmodium gallinaceum* was detected from the chickens<br>- Infections were more in 8 % females than males (5 %)<br>- Highest prevalence was observed in chickens with packed cell volume (PCV) range of 21-25 had the highest prevalence (38.46 %), followed by those with PCV range of 16-20 (30.76 %) | Nigeria |
| Opara et al., 2012 | To determine the blood characteristics, prevalence of gastro-intestinal and haemoparasites of street pigeons in Owerri. | Pigeons | *Haemoproteus* spp., *Plasmodium* spp. | - *Plasmodium* spp. and *Haemoproteus* spp were detected<br>- *Haemoproteus* spp was the most dominant compared to *Plasmodium* spp.<br>- 80 % (120 of 150) of pigeons tested positive<br>- Infection being high in female chickens (66.7 %) than in male chickens (33.3 %) | Nigeria |
| Usman et al., 2012 | To determine the prevalence of ecto and haemoparasites of chickens in Sokoto metropolis. | Chickens | *Plasmodium gallinaceum* | - Only one species of haemoparasites was detected from the examined free-range chickens, *Plasmodium gallinaceum*<br>- *Plasmodium gallinaceum* was detected from 12 of 100 free range chickens. | Nigeria |
| Sabuni et al., 2011 | To determine the type and prevalence of haemoparasites infection in indigenous free-ranging village chickens in two different agro-ecological zones in Kenya. | Chickens | *Plasmodium gallinaceum*, *Leucocytozoon schoutedeni*, *Haemoproteus* spp. | -*Plasmodium gallinaceum*, *Leucocytozoon schoutedeni* and *Haemoproteus* spp. were found infesting poultry<br>-*Plasmodium gallinaceum* was the most and *Haemoproteus* spp. was the least encountered haemoparasite infesting chickens<br>- 71 (62.3 %) of infected chickens had single infection, while 43 (37.7 %) were infected by more than one genus of haemoparasites<br>- *Leucocytozoon schoutedeni* showed an increase in prevalence rate with increase in age of the chicken. | Kenya |
| Msoffe et al., 2010 | To gauge the occurrence of parasites (endoparasites, ectoparasites and blood) of pigeons in Morogoro Municipality of Tanzania. | Pigeons | *Haemoproteus columbae* | - Both nesting and adult pigeons were infected by the haemoparasite species, *Haemoproteus columbae*.<br>- Results showed that, *Haemoproteus columbae* was higher in adult pigeons compared to the nesting pigeons. | Tanzania |
| Igbokwe et al., 2008 | - | Chickens and | *Plasmodium* spp. | - *Plasmodium* was the only detected haemoparasite in chickens and Guinea fowls | Nigeria |

| | | | | | |
|---|---|---|---|---|---|
| | | guinea fowls | | - Infection observed in domestic chickens and Guinea fowls suggested that avian malaria was prevalent in this environment<br>- Infections increased with increasing rainfall<br>- *Plasmodium* infection in chickens did not decrease their PCV | |
| Sehgal et al., 2006 | To study the prevalence and morphology of 2 distinct morphospecies of the chicken blood parasites *L. schoutedeni* and *T. gallinarum*, and assign DNA sequence information to them. | Chickens | *Leucocytozoon schoutedeni* | - Chickens were infected by *Leucocytozoon schoutedeni* only<br>- The chickens from this study were collected from several habitats where biting midges and mosquitoes occurs in high numbers.<br>- Therefore, absence of *Plasmodium* and *Haemoproteus* spp. infections of these birds was unexpected. | Cameroon and Uganda |
| Illango et al., 2002 | To develop integrated and appropriate health management interventions targeting rural poultry production in two agro-ecological zones of Uganda | Chickens | *Plasmodium* spp. | - The blood smears indicated the presence of only *Plasmodium* spp. in both dry and wet seasons. | Uganda |
| Permin et al., 2002 | To describe the prevalences of parasitic infections and infestations in free-range village chickens in the Goromonzi District in Zimbabwe. | Chickens | *Leucocytozoon sabrazesi, Plasmodium gallinaceum, Trypanosoma avium* | - 32% were infected with haemoparasites.<br>- Prevalence's (%) of haemoparasites in young and adult chickens were: *Leucocytozoon sabrazesi* (3; 1), *Plasmodium gallinaceum* (8; 6) and *Trypanosoma avium* (2; 3), respectively. | Zimbabwe |
| Mushi et al., 2000 | To determine the presence of endo, ecto- and haemoparasites of pigeons, some of which may be of zoonotic importance. | Pigeons | *Haemoproteus columbae* | - *Haemoproteus columbae* was the most common blood parasite found on pigeons.<br>- The parasite was not associated with clinical disease. | Botswana |
| Poulsen et al., 2000 | To determine the species and their prevalences of gastro-intestinal helminths and haemoparasites in scavenging chickens in the upper eastern region of Ghana, West Africa. | Chickens | *Plasmodium juxtanucleare* | - The parasites collected in this study was *Plasmodium juxtanucleare*<br>- It was surprising that *Leucocytozoon* species were not detected in domestic chickens studied, as it is commonly found in the blood of domestic chickens | Ghana |
| Dranzoa et al., 1999 | To determine the prevalence of ecto-, gastro-intestinal- and haemo-parasites of pigeons. | Pigeons | *Haemoproteus* spp.<br>*Plasmodium* spp. | - *Haemoproteus* spp. and *Plasmodium* spp. were the only blood parasites infecting pigeons<br>- *Haemoproteus* spp. was the most abundant specie (76.5 %) than *Plasmodium* spp. (29.4 %)<br>- Mixed infections by *Haemoproteus* spp. and *Plasmodium* spp. were detected in 10 pigeons | Uganda |

| | | | | | |
|---|---|---|---|---|---|
| Mushi et al., 1999 | - | Pigeons | *Haemoproteus columbae* | -The erythrocytes of high proportion (75 %) of health pigeons were parasitized by *H. columbae* gametes.<br>- The blood parasite was identified as *H. columbae.*<br>- The high prevalence could be attributed to the presence of blood sucking vector flies. | Botswana |
| Earle, 1993a | To investigate inter-specificity and intraspecific host-parasite and distributions of avian haematozoan in various game birds in southern Africa | Guinea fowls | *Haemoproteus pratasi, Leucocytozoon neavei* | - The overall infection rate of haemoparasites in guinea fowls was 52.2 %<br>- The species *Haemoproteus pratasi* and *Leucocytozoon neavei* were present in 3 different sampling locations | Zambia and Zimbabwe |
| Earle, 1993b | To determine which blood parasites occurred in mixed flocks of feral and rock pigeons and to investigate if prevalence of parasite infection or parasite burdens varied between those two closely related but ecologically differentiated, host species. | Pigeons | *Haemoproteus columbae, Leucocytozoon marchouxi* | - *Haemoproteus columbae* was the most abundant species infecting pigeons<br>- The infection of *Haemoproteus columbae* was greater in females than in males only in feral pigeons<br>- The infection of *Haemoproteus columbae* was independent of host-sex in the rock pigeons.<br>- *Haemoproteus columbae* was higher in feral pigeons (73 %) as compared to rock pigeons (12 %) | South Africa |
| Earle et al., 1991 | - | Guinea fowls | *Haemoproteus pratasi, Hepatozoon* spp., *Leucocytozoon neavei, Plasmodium circumflexum, Trypanosoma numidae* | - Guinea fowls were infected by parasites from five families and genera of haemoparasites.<br>- *Leucocytozoon neavei* was the most abundant parasite (56 %), followed by *Haemoproteus pratasi* (49%).<br>- *Plasmodium circumflexum* (3.5 %), *Trypanosoma numidae* (2.5 %) and Hepatozoon sp. (1.0 %) were recorded in less numbers. | South Africa |
| Fallis et al., 1973 | - | Chickens and guinea fowls | *Leucocytozoon schoutedeni* | - More than 50 % of the examined chickens were infected by *Leucocytozoon schoutedeni*<br>- Blood smears from guinea fowls showed the presence of *Leucocytozoon schoutedeni* | Tanzania |
